# Exploring the associations between dietary nitrate and nitrite intake and markers of self-reported periodontal disease in the UK Biobank cohort

**DOI:** 10.1007/s00394-026-04035-2

**Published:** 2026-06-29

**Authors:** Shatha S. Alhulaefi, Anthony Watson, Kirsten Brandt, Sheena E. Ramsay, Nicholas S. Jakubovics, Alex Griffiths, Jamie Matu, Mario Siervo, Benjamin H. Parmenter, Liezhou Zhong, Jonathan Hodgson, Catherine P. Bondonno, Nicola P. Bondonno, Oliver M. Shannon

**Affiliations:** 1 Human Nutrition & Exercise Research Centre, Centre for Healthier Lives, Population Health Sciences Institute, Newcastle University, Newcastle Upon Tyne, UK; 2 Department of Nutrition, Faculty of Science, Taif University, P.O. Box 11099, 21944 Taif, Saudi Arabia; 3 Population Health Sciences Institute, Newcastle University, Newcastle upon Tyne, UK; 4 Faculty of Medical Sciences, School of Dental Sciences, Newcastle University, Newcastle upon Tyne, UK; 5 School of Health, Leeds Beckett University, Leeds, UK; 6 School of Population Health, Curtin University, Perth, WA Australia; 7 Dementia Centre of Excellence, enAble Institute, Curtin University, Perth, Australia; 8 Curtin-Chulalongkorn Collaborative Centre for Nutrition and Food Research and Education, Curtin University, Perth, Australia; 9 Curtin Medical School, Curtin Medical Research Institute, Curtin University, Bentley, WA Australia; 10 School of Medical and Health Sciences, Nutrition and Health Innovation Research Institute, Edith Cowan University, Perth, Australia; 11 Institute of Agriculture, The University of Western Australia, Perth, WA Australia; 12 The Danish Cancer Institute, Copenhagen, Denmark; 13 Co-Centre for Sustainable Food Systems and Institute for Global Food Security, Queen’s University Belfast, Belfast, UK

**Keywords:** Dietary nitrate, UK Biobank, Periodontal disease, Diet

## Abstract

**Purpose:**

Periodontal disease, an inflammatory condition affecting the tissue surrounding the teeth, has been associated with various systemic health issues. Dietary nitrate and nitrite are found in a range of plant and animal foods and, depending upon source, have been linked with both positive and negative health effects, including improved oral health with plant sources. This study aimed to investigate the associations between source-dependent (plant- and animal-sourced) nitrate and nitrite intake and odds of periodontal disease.

**Methods:**

We explored cross-sectional and longitudinal associations between tertiles of source-dependent nitrate and nitrite intake and the odds of periodontal disease using multivariable logistic regression models (cross-sectional analyses) and generalised estimating equations (longitudinal analyses) in 158,778 and 83,026 participants, respectively, from the UK Biobank. Dietary nitrate/nitrite intake was estimated from 24 h dietary assessments and a comprehensive food composition database.

**Results:**

Higher intake of plant-derived nitrate was associated with significantly lower odds of periodontal disease in cross-sectional (OR [95%CI]: 0.925 [0.894–0.958]) and longitudinal (OR [95%CI]: 0.880 [0.840–0.921]) analyses compared with lower intakes. A similar pattern of association was observed for plant-derived nitrite. Animal-derived nitrate intake was not associated with odds of periodontal disease. Higher intake of animal-derived nitrite was associated with significantly higher odds of periodontal disease in cross-sectional analyses only (OR [95% CI]: 1.043 [1.009–1.078]).

**Conclusion:**

Plant-derived nitrate and nitrite were associated with lower odds of self-reported periodontal disease, whilst animal-derived nitrite showed adverse associations in cross-sectional analyses only. Associations between dietary nitrate and nitrite with oral health outcomes may therefore depend on food source. Further mechanistic and intervention research is needed to test causality.

**Supplementary Information:**

The online version contains supplementary material available at 10.1007/s00394-026-04035-2.

## Introduction

Oral diseases, including periodontal disease, consistently rank as the most prevalent health condition globally, affecting approximately 3.69 billion people worldwide [1]. Periodontal disease is a chronic inflammatory condition that impacts the tissues and bones that support teeth and includes gingivitis and periodontitis [2]. Gingivitis is the initial stage of this condition, which causes pain, swelling, and gingival bleeding. This then develops into periodontitis, the most advanced stage which can lead to tooth loss [2]. Periodontal disease is associated with a higher risk of other health concerns, including diabetes [3], cardiovascular disease [4], and head and neck cancer [5], inflammatory arthritides [6] and chronic obstructive pulmonary disease [7]. Therefore, preventing/treating this condition could have implications for health beyond the oral cavity. Periodontal disease also has a large economic burden, costing approximately €159 billion across 32 European countries and $154 billion in the United States of America, respectively, in 2018 [8]. This underscores the importance of identifying effective strategies to reduce risk of this condition.

Dietary nitrate is an inorganic anion present in a wide range of plant and animal food sources alongside drinking water [9, 10]. Green leafy vegetables are a particularly significant source of nitrate, contributing up to 80% of total nitrate intake [11]. Over the past two decades, research has identified that consumption of dietary nitrate can lead to positive effects on a plethora of health outcomes, lowering blood pressure [12–14], decreasing platelet aggregation [15–17], enhancing endothelial function [18, 19], and improving exercise tolerance/ performance [20–22]. These beneficial effects appear to be related to the conversion of nitrate into nitrite and subsequently nitric oxide (NO)—a multifunctional gasotransmitter—via the nitrate-nitrite-NO pathway [23]. The conversion of nitrate into nitrite is largely dependent upon bacteria in the mouth, which use nitrate as an alternative electron accepter during respiration [24]. The mouth is therefore an important site for nitrate ‘processing’ in the body. Interestingly, recent data also suggests that consumption of dietary nitrate can have favourable effects on oral health. For example, data from recent systematic reviews suggests that nitrate consumption enhances the abundance of bacteria associated with oral health and reduces the abundance of bacteria that cause oral diseases [25, 26]. Furthermore, studies have shown that consuming nitrate reduces salivary acidity, salivary lactate [27, 28] and the gingival inflammation index[29]. However, to date, no epidemiological studies have investigated the association between nitrate intake and the odds of periodontal disease in a large population sample.

It has been suggested that the health effects of dietary nitrate might differ depending upon whether nitrate is consumed from plant or animal sources [30, 31]. For example, Bondonno et al. [32] found that higher intake of nitrate from plant sources is associated with a lower risk of all-cause mortality, whilst higher intake of nitrate from animal sources was associated with a higher mortality. Similar findings were observed in another recent study that investigated the association between dementia-related mortality risk and source-dependent nitrate intake[33]. Mechanistically, this could be related to increased generation of NO and lower generation of potentially harmful *N*-nitrosamines with plant versus animal sources of nitrate [31]. However, more research is needed to investigate the impact of source on the health effects of nitrate. Therefore, this study aims to investigate the cross-sectional and longitudinal associations between source-dependent dietary nitrate/ nitrite intake (plant/animal sources) and odds of periodontal disease in participants from the UK Biobank cohort.

## Methods

### Study design and population

This study explored associations between dietary nitrate intake from plant/animal sources and the odds of periodontal disease using data from the UK Biobank, a population-based cohort study with ethical approval from the North West Haydock Research Ethics Committee (Ref: 16/NW/0274). Details of the study design and data collection methods are presented in a previously published paper [34]. However, briefly, the UK Biobank recruited over half a million participants aged 40 to 69 years across Great Britain using National Health Service (NHS) patient records between 2006 and 2010. After providing signed electronic consent, the participants visited one of 22 assessment centres across the UK. Here, they each completed a self-administered touchscreen questionnaire and short interview and underwent physical function and anthropometric assessments. The researchers also collected blood, saliva and urine samples from the participants. Participants were later invited to undergo additional tests, including detailed dietary assessments and whole-body imaging. Our study included both cross-sectional and longitudinal analyses. For the cross-sectional analysis, the analytical sample included participants who self-reported oral health status at the baseline assessment-centre visit, completed at least one Oxford WebQ dietary assessment (outline further below) used to estimate habitual dietary nitrate and nitrite intake, and had complete data for relevant baseline covariates. For the longitudinal analysis, participants were included if they completed at least one Oxford WebQ dietary assessment, provided relevant baseline covariate data, and self-reported oral health status at one or more timepoints on or after their first dietary assessment. Baseline assessment-centre visits occurred between 2006 and 2010; however, because the Oxford WebQ was introduced part-way through this period and was also administered online at later timepoints, dietary assessment was not always completed contemporaneously with baseline oral health assessment. Therefore, oral health observations recorded before the first available Oxford WebQ did not contribute to the longitudinal analyses. This ensured that periodontal disease outcomes included in the longitudinal analysis did not precede the first dietary assessment.

### Dietary assessment

Diet was evaluated in the UK Biobank via a basic touchscreen questionnaire and a more comprehensive 24-h dietary assessment tool (Oxford WebQ) that has been validated for use in observational research [35, 36]. For this study, we used dietary data from the Oxford WebQ to estimate dietary nitrate intake (see ‘Calculation of dietary nitrate and nitrite intakes’). This tool collects data on the consumption of 206 different types of foods and 32 drinks by determining the number of standard portions that were consumed for each item. Participants completed up to five nutritional assessments using this tool. Participants who conducted their baseline visit between April 2009 and September 2010 completed the Oxford WebQ as part of their assessment centre visits (dietary assessment 1). Dietary data were not collected at baseline using this tool from participants who visited their assessment centres prior to these dates. All participants who provided email addresses were invited to complete a nutritional assessment using this tool at home on their computers every three to four months between February 2011 and June 2012 (dietary assessments 2–5). For this analysis, self-reported atypical dietary reports were not used. In our study sample, the number of participants who completed dietary questionnaires was as follows: in the cross-sectional analysis, 55,694, 37,789, 34,933, 25,517, and 4,845 participants completed one, two, three, four, and five questionnaires, respectively. The corresponding numbers in the longitudinal analysis were 30,320, 17,232, 16,791, 13,838, and 4,845, respectively.

### Calculation of dietary nitrate and nitrite intakes

Dietary data from the Oxford WebQ were used to calculate average nitrate and nitrite intakes. Nitrate intake values were quantified using the Nitr-Navigator Food Composition Database (version 2023v0.0.6 [https://nitr-navigator.com/]). This database contains nitrate and nitrite content data for over 800 foods and beverages including from plant and animal sources from over 80 countries [9, 10]. As the nitrate and nitrite content of foods varies according to the country of cultivation, the following strategy was employed: for each food, if three or more entries were available in the database for the United Kingdom, the median of these values was used; if there were less than three entries in the database for the United Kingdom, the median of values for all Europe were used; if there were less than three entries available for Europe, the median value from all countries in the database were used. The food codes from the updated UK Nutrient Databank [37] were used to match the foods and beverages in the Oxford WebQ with the most similar food in the Nitr-Navigator Database, based on food names and processing and preparation methods. Due to the effect of cooking and boiling on nitrate/nitrite content [38], a 50% reduction in nitrate and nitrite values for cooked vegetables was applied [39]. The nitrate/nitrite content of each food (mg/g) was multiplied by the intake reported in Oxford WebQ (g/day) and then summed across all foods and drinks consumed for each participant to calculate daily nitrate and nitrite intakes (mg/day) using facilities at the Pawsey Supercomputing Centre (Perth, Australia).

Overall, nitrate and nitrite intake by food source was calculated for 12 food groups (Table S1). For each participant, nitrate and nitrite intake data from each food group, as described above, were calculated at each time point. Participants who completed the Oxford WebQ assessment but reported no nitrate or nitrite intake were given a zero value, while those who reported an atypical diet were excluded from that specific time point. The mean nitrate and nitrite intake was then calculated as the arithmetic mean of all available valid time points for each participant. In this study, the main exposures to nitrate and nitrite were identified as plant sources (including from fruit, green leafy vegetables, potatoes, other vegetables, wholegrains and nuts) and animal sources (including meat, fish, other animal sources and processed meat). The subgroups created for use in sensitivity analyses were vegetable-derived nitrate/ nitrite (potatoes, green leafy vegetables and other vegetables), unprocessed animal-derived nitrate/ nitrite (meat, fish and other animal sources) and processed meat-derived nitrate/ nitrite (processed meats). Unprocessed meats naturally contain nitrate and nitrite, and nitrate intake from unprocessed meat sources has been reported to be tenfold higher than from processed meats [40]. We classified animal sources into unprocessed and processed meats to assess whether the associations between total animal-derived nitrate and nitrite were due to processed meats, where nitrate and nitrite are added as preservatives, or to naturally occurring nitrate and nitrite in unprocessed meat. This approach has also been used in previous studies [32].

### Periodontal disease

Participants reported their oral health status via a touch-screen questionnaire on four separate occasions from 2006 to 2019 (the initial assessment visit, the first repeat assessment visit, the imaging visit and the first repeat imaging visit). They were required to self-report whether they had one or more of the following oral health outcomes over the last 12 months: denture use, toothache, mouth ulcers, bleeding gums, gum pain, loose teeth or none of the above. None of these occasions included a clinical oral examination. Participants were considered at increased risk for periodontal disease if they reported one or more of the following symptoms: bleeding gums, painful gums or loose teeth. Bleeding and painful gums have been associated with mild to moderate periodontal disease, and loose teeth signify severe periodontal disease [41]. Therefore, these self-reported symptoms were considered an indirect indicator of periodontal disease, as they have been shown to be valid without a clinical diagnosis [42, 43]. Participants who did not report these symptoms were considered at low risk of periodontal disease. This approach has been used in previous analyses in UK Biobank [6, 44–46].

### Covariate assessment

Data on age, sex, physical activity, education, alcohol consumption, and smoking status were obtained through self-reported questionnaires. The Townsend Deprivation Index (TDI), an area-based measure of socioeconomic deprivation, was based on participants’ postcodes of residence [47]. During the baseline assessment visit, height (cm) and weight (kg) were measured in all participants to calculate body mass index (BMI). The above variables were included as covariates in the present analyses and categorised as per previous research [48] as follows: alcohol consumption (never, previous, or current), smoking status (never, previous, and current), physical activity (low, moderate, or high), educational level (higher, vocational, upper secondary, lower secondary, or other), TDI score (low, medium, or high) BMI (underweight [< 18 kg/m^2^], healthy weight [≥ 18 to < 25 kg/m^2^], overweight [25 to < 30 kg/m^2^], and obese [> 30 kg/m^2^]). Since the majority of participants were of White ethnicity, the ethnicity variable was reclassified into two categories to ensure appropriate group sizes for statistical analysis. Participants were categorised as either (1) White or (2) Other ethnic groups, which included Mixed, Asian or Asian British, Black or Black British, Chinese, or any other ethnic backgrounds [44]. All covariates were measured during the baseline assessment visit and were used in both cross-sectional and longitudinal analyses.

### Statistical analysis

Data analyses were conducted using SPSS version 29.0 for Windows. Demographic variables and baseline characteristics were presented as means ± standard deviation for continuous variables and percentages for categorical variables based on dietary nitrate/ nitrite intake (low, moderate, or high). Logistic regression models were utilised to assess the cross-sectional associations between tertiles of dietary nitrate/nitrite intake by source (plant/animal) and odds of periodontal disease at baseline. Average nitrate and nitrite intake by source (plant/animal) were entered separately into each model as an independent variable after being divided into approximately equal tertiles, with the lowest nitrate and nitrite intake groups used as the reference category, consistent with previous studies [49, 50] Self-reported periodontal disease risk was determined by the presence of symptoms defined for this study (see the “Periodontal disease” section) versus the absence of these symptoms and entered into each model as a binary dependent variable. Covariates were entered simultaneously into each model using the enter method. To assess the longitudinal associations between nitrate and nitrite intake and the odds of periodontal disease across all collected time points, generalised estimating equations (GEE) were used. An exchangeable correlation structure was identified for the data with the lowest quasi-likelihood under the independence model criterion (QIC) value, which indicated the best fit for the statistical model. The results of the analyses were presented as odds ratios (ORs) and 95% confidence intervals (CIs) for each group compared to a low intake reference group. For the GEE analyses the ORs should be interpreted as the population-averaged odds of periodontal disease across the study follow up period (~ 10 years). Three models were used for all analyses as follows: Model 1: adjusting for age and sex; Model 2: Model 1 adjustments plus BMI, smoking status, alcohol drinking status, physical activity, socioeconomic status, ethnicity, and educational level; Model 3: If nitrate or nitrite from a plant source were the exposures of interest, intakes of poultry, unprocessed meat, processed meat, fish, dairy products, refined grains, coffee, tea, soft drinks, sugar and confectionary were included in addition to the covariates included in Model 2. If animal-derived nitrate or nitrite were the exposures of interest, intakes of fruits, vegetables, whole grains, refined grains, tea, coffee, soft drinks, sugar and confectionery were included, along with the covariates included in Model 2.

Considering associations might differ between population groups, as the literature has shown that sex, age, smoking status, and BMI are associated with periodontal disease risk [51–53], we conducted stratified analyses using the cross-sectional dataset to detect any between-group differences as follows: older adults vs. young adults (based on the median age), female vs male, current or former smokers vs. non-smokers, healthy BMI [≥ 18 to < 25 kg/m^2^] vs. overweight and obesity [> 25 kg/m^2^]. These analyses were focused on the baseline cross-sectional data, as this ensures that exposure, outcome, and stratification variables were measured concurrently. Some stratifying covariates (e.g., smoking status, BMI) are time-varying and may have changed over the follow-up period, such that using longitudinal data could complicate subgroup definitions and interpretation of effect modification.

### Sensitivity analyses

Multiple sensitivity analyses were conducted to assess the robustness of the associations between source-specific nitrate/nitrite intake and the odds of periodontal disease. Firstly, analyses were replicated using three sub-groups of nitrate/nitrite sources (vegetables, unprocessed meats, and processed meats). Secondly, analyses were repeated after dividing the intake groups into quartiles and quintiles, rather than tertiles. Thirdly, for the purpose of providing a more accurate measure of habitual dietary intake, the analyses were repeated after only including participants who provided at least two reports and, separately, after excluding dietary reports indicating extreme energy intakes (over 4,200 or less than 800 kcal/d for male participants and over 3,500 or less than 600 kcal/d for female participants) [48]. Fourthly, to examine whether overall diet quality impacted associations between nitrate and nitrite intake and self-reported periodontal disease risk, analyses were repeated after including a composite diet quality score reflective of UK healthy eating guidelines (Eatwell guide score)[54] as a covariate instead of individual food items. Fifthly, to examine whether temporal asynchrony between periodontal disease measurement and dietary reporting affected cross-sectional associations, analyses were repeated after excluding participants whose periodontal disease was measured at the baseline visit prior to their first Oxford WebQ completion. Finally, analyses were conducted exploring associations between nitrate and nitrite intake and all the individual oral health markers reported in the UK Biobank Oral Health Questionnaire (denture use, toothache, mouth ulcers, bleeding gums, gum pain, loose teeth) to see if any specific oral health changes were driving the associations with the composite measure of periodontal disease.

## Results

### Cohort characteristics

Our cross-sectional analysis included 158,778 participants out of the 502,536 who underwent baseline assessment of the UK Biobank study, while the longitudinal analysis included 83,026 participants (Fig. S1 in the supplementary material). Baseline demographic and health characteristics for participants included in the cross-sectional analysis, stratified by level of plant and animal nitrate intake, are presented in Table 1. Participants had a mean age of 55.9 ± 7.9 years, and over 95% were White. The number of participants who showed symptoms of periodontal disease was 27,095 (17.1%). The longitudinal analysis was based on participant data across all visits, with data available for at least 41,000 participants on two or more occasions. The median nitrate intake in the cross-sectional analyses from plant sources was 70.30 mg/day (range: 0 to 1368.29 mg/day). The median nitrate intake from animal sources was 3.27 mg/day (range: 0 to 42.15 mg/day). The wide range reflects considerable variability in intake among individuals. Leafy green vegetables were the main contributor to nitrate intake (32.0 mg/day, equivalent to ~ 35% of the total), followed by other vegetables such as cruciferous and root vegetables (24 mg/day, ~ 27%), and then fruits (18 mg/day, ~ 20%).

**Table 1:** Characteristics participants of the study sample of the UK Biobank

	Total *(n* = *158,778)*	Plant-based nitrate intake			Animal-based nitrate intake		
		Low (≤ 49.19 mg/d) *(n* = *51,550)*	Medium (49.20–95.83 mg/d) *(n* = *53,335)*	High (≥ 95.84 mg/d) *(n* = *53,893)*	Low (≤ 2.23 mg/d) *(n* = *51,995)*	Medium (2.24–4.15 mg/d) *(n* = *52,980)*	High (≥ 4.16 mg/d) *(n* = *53,803)*
Age							
(mean ± SD), years	55.9 ± 7.9	55.0 ± 8.1	56.23 ± 7.9	56.71 ± 7.8	55.38 ± 8.1	56.09 ± 7.9	56.49 ± 7.9
Sex							
Male	74,775 (47.1%)	29,840 (57.9%)	25,200 (47.2%)	19,735 (36.6%)	25,544 (49.1%)	24,970 (47.1%)	24,261 (45.1%)
Female	84,003 (52.9%)	21,710 (42.1%)	28,135 (52.8%)	34,158 (63.4%)	26,451 (50.9%)	28,010 (52.9%)	29,542 (54.9%)
Ethnicity							
White	151,946 (95.7%)	49,005 (95.1%)	51,160 (95.9%)	51,781 (96.1%)	49,182 (94.6%)	51,022 (96.3%)	51,742 (96.2%)
Other ethnic groups	6410 (4.0%)	2400 (4.7%)	2042 (3.8%)	1968 (3.7%)	2660 (5.1%)	1839 (3.5%)	1911 (3.6%)
BMI (kg/m^2^)							
< 18	459 (0.3%)	115 (0.2%)	156 (0.3%)	188 (0.3%)	181 (0.3%)	151 (0.3%)	127 (0.2%)
≥ 18– < 25	58,328 (36.7%)	16,137 (31.3%)	20,388 (38.2%)	21,803 (40.5%)	19,931 (38.3%)	19,354 (36.5%)	19,043 (35.4%)
25–29.9	65,255 (41.1%)	22,222 (43.1%)	21,940 (41.1%)	21,093 (39.1%)	20,901 (40.2%)	22,054 (41.6%)	22,300 (41.4%)
≥ 30	34,736 (21.9%)	13,076 (25.4%)	10,851 (20.3%)	10,809 (20.1%)	10,982 (21.1%)	11,421 (21.6%)	12,333 (22.9%)
Education							
Higher	95,388 (60.1%)	27,127 (52.6%)	33,139 (62.1%)	35,122 (65.2%)	30,556 (58.8%)	31,543 (59.5%)	33,289 (61.9%)
Vocational	18,243 (11.5%)	7311 (14.2%)	5734 (10.8%)	5198 (9.6%)	6144 (11.8%)	6162 (11.6%)	5937 (11.0%)
Upper secondary	10,136 (6.4%)	3359 (6.5%)	3483 (6.5%)	3294 (6.1%)	3286 (6.3%)	3453 (6.5%)	3397 (6.3%)
Lower secondary	22,820 (14.4%)	8728 (16.9%)	7254 (13.6%)	6838 (12.7%)	7673 (14.8%)	7730 (14.6%)	7417 (13.8%)
Other	12,191 (7.7%)	5025 (9.7%)	3725 (7.0%)	3441 (6.4%)	4336 (8.3%)	4092 (7.7%)	3763 (7.0%)
Socioeconomic status ^a^							
1 (least deprived)	34,615 (21.8%)	10,953 (21.2%)	11,924 (22.4%)	11,738 (21.8%)	10,667 (20.5%)	11,840 (22.3%)	12,108 (22.5%)
2–4	98,300 (61.9%)	31,634 (61.4%)	33,229 (62.3%)	33,437 (62.0%)	31,898 (61.3%)	32,903 (62.1%)	33,499 (62.3%)
5 (most deprived)	25,863 (16.3%)	8963 (17.4%)	8182 (15.3%)	8718 (16.2%)	9430 (18.1%)	8237 (15.5%)	8196 (15.2%)
Smoking status							
Never	89,855 (56.6%)	28,271 (54.8%)	30,925 (58.0%)	30,659 (56.9%)	28,968 (55.7%)	30,265 (57.1%)	30,622 (56.9%)
Previous	56,642 (35.7%)	17,857 (34.6%)	18,875 (35.4%)	19,910 (36.9%)	18,404 (35.4%)	18,828 (35.5%)	19,410 (36.1%)
Current	12,019 (7.6%)	5325 (10.3%)	3456 (6.5%)	3238 (6.0%)	4532 (8.7%)	3792 (7.2%)	3695 (6.9%)
Physical activity levels							
Low (least active)	28,520 (18.0%)	11,501 (22.3%)	9378 (17.6%)	7641 (14.2%)	9616 (18.5%)	9598 (18.1%)	9306 (17.3%)
Moderate	66,986 (42.2%)	21,672 (42.0%)	23,179 (43.5%)	22,135 (41.1%)	21,902 (42.1%)	22,439 (42.4%)	22,645 (42.1%)
High (most active)	63,272 (39.8%)	18,377 (35.6%)	20,778 (39.0%)	24,117 (44.7%)	20,477 (39.4%)	20,943 (39.5%)	21,852 (40.6%)
Alcohol drinker status							
Never	4850 (3.1%)	1595 (3.1%)	1609 (3.0%)	1646 (3.1%)	1765 (3.4%)	1574 (3.0%)	1511 (2.8%)
Previous	4790 (3.0%)	1650 (3.2%)	1465 (2.7%)	1675 (3.1%)	1709 (3.3%)	1578 (3.0%)	1503 (2.8%)
Current	149,076 (93.9%)	48,282 (93.7%)	50,239 (94.2%)	50,555 (93.8%)	48,499 (93.3%)	49,809 (94.0%)	50,768 (94.4%)
Risk of periodontal disease							
	27,095 (17.1%)	9307 (18.1%)	8866 (16.6%)	8922 (16.6%)	9036 (17.4%)	8829 (16.7%)	9230 (17.2%)

a; Categories derived from the Townsend Index of Deprivation were used to indicate socioeconomic status, with quintiles 1 = low (least deprived), 2–4 = medium, 5 = high (most deprived)

Participants in the highest tertile of plant-sourced nitrate intake tended to be female, with a higher level of education, a healthy BMI, had never smoked, and were more physically active than participants in the lower tertile. Furthermore, the number of individuals reporting periodontal disease symptoms in the higher tertile of plant-sourced nitrate intake was lower compared with the bottom tertile. Similar trends were observed for animal-sourced nitrate, though differences were less pronounced between the highest and lowest tertiles.

### Associations between plant-sourced nitrate and nitrite and self-reported risk of periodontal disease

Cross-sectionally, moderate and high intakes of plant sourced-nitrate were associated with 5.9% and 7.5% lower odds of periodontal disease, respectively [OR (95% CI), Moderate: 0.941 (0.911–0.973), High: 0.925 (0.894–0.958); Model 3, Table 2], when compared to low intakes. Comparable associations [OR (95%CI)] were seen in longitudinal analyses [Moderate: 0.879 (0.841–0.919), High: 0.880 (0.840–0.921); Model 3, Table 3]. Associations [OR (95%CI)] for plant-sourced nitrite were slightly stronger both cross-sectionally [Moderate: 0.883 (0.858–0.910), High: 0.862 (0.826–0.900); Model 3, Table 2] and longitudinally [Moderate: 0.866 (0.832–0.901), High: 0.865 (0.817–0.917); Model 3, Table 3].

**Table 2:** Cross-sectional associations between nitrate/nitrite intakes and odds (95% CI) of periodontal disease in the UK Biobank study

	Lower intake (≤ 49.19 mg/d)	Moderate intake (49.20–95.83 mg/d)	Higher intake (≥ 95.84 mg/d)
Total plant-based nitrate			
Model 1^a^ (n = 158,778)	Reference	0.896 (0.868–0.926) **	0.872 (0.844–0.901) **
Model 2^b^ (n = 158,778)	Reference	0.933 (0.903–0.964) **	0.913 (0.883–0.944) **
Model 3^c^ (n = 158,778)	Reference	0.941 (0.911–0.973) **	0.925 (0.894–0.958) **

	Lower intake (≤ 2.23 mg/d)	Moderate intake (2.24–4.15 mg/d)	Higher intake (≥ 4.16 mg/d)
Total animal-based nitrate			
Model 1^a^ (n = 158,778)	Reference	0.958 (0.928–0.990) *	0.995 (0.964–1.028)
Model 2^b^ (n = 158,778)	Reference	0.973 (0.942–1.005)	1.009 (0.977–1.042)
Model 3^c^ (n = 158,778)	Reference	0.980 (0.949–1.013)	1.024 (0.992–1.058)

	Lower Intake (≤ 1.30 mg/d)	Moderate intake (1.31–2.49 mg/d)	Higher intake (≥ 2.50 mg/d)
Total plant-based nitrite			
Model 1^a^ (n = 158,778)	Reference	0.842 (0.818 – 0.867) **	0.817 (0.783–0.852) **
Model 2^b^ (n = 158,778)	Reference	0.880 (0.854 – 0.906) **	0.860 (0.824–0.897) **
Model 3^c^ (n = 158,778)	Reference	0.883 (0.858 – 0.910) **	0.862 (0.826–0.900) **

	Lower intake (≤ 0.44 mg/d)	Moderate intake (0.45–0.73 mg/d)	Higher intake (≥ 0.74 mg/d)
Total animal-based nitrite			
Model 1^a^ (n = 158,778)	Reference	0.969 (0.938–1.000)	1.056 (1.023–1.090) **
Model 2^b^ (n = 158,778)	Reference	0.986 (0.955–1.019)	1.049 (1.016–1.084) *
Model 3^c^ (n = 158,778)	Reference	0.988 (0.956–1.020)	1.043 (1.009–1.078) *

a; adjusted for age and sex, b; adjusted for age, sex, ethnicity, BMI, smoking, alcohol intake, educational level, socioeconomic status (Townsend deprivation index), physical activity level, c; adjusted for the covariates in Model 2 and intakes of (1) processed meat, red meat, poultry, fish, dairy, refined grains, coffee, tea, soft drinks, and sugar and confectionary when plant-sourced nitrate or nitrite were the exposures of interest, and (2) vegetables, fruits, wholegrains, refined grains, coffee, tea, soft drinks, and sugar and confectionary when animal-sourced nitrate or nitrite were the exposures of interest., *; *p* < 0.05, **; *p* < 0.001

**Table 3:** Longitudinal associations between nitrate/nitrite intakes and odds (95% CI) of periodontal disease in the UK Biobank study

	Lower Intake (≤ 49.19 mg/d)	Moderate Intake (49.20–95.83 mg/d)	Higher Intake (≥ 95.84 mg/d)
Total plant-based nitrate			
Model 1^a^ (n = 83,026)	Reference	0.822 (0.788–0.859) **	0.811 (0.777–0.848) **
Model 2^b^ (n = 83,026)	Reference	0.868 (0.830–0.906) **	0.863 (0.826–0.903) **
Model 3^c^ (n = 83,026)	Reference	0.879 (0.841–0.919) **	0.880 (0.840–0.921) **

	Lower intake (≤ 2.23 mg/d)	Moderate intake (2.24–4.15 mg/d)	Higher intake (≥ 4.16 mg/d)
Total animal-based nitrate			
Model 1^a^ (n = 83,026)	Reference	0.958 (0.917–1.000)	0.992 (0.950–1.036)
Model 2^b^ (n = 83,026)	Reference	0.978 (0.936–1022)	1.014 (0.971–1.059)
Model 3^c^ (n = 83,026)	Reference	0.987 (0.945–1.031)	1.032 (0.988–1.078)

	Lower intake (≤ 1.30 mg/d)	Moderate intake (1.31–2.49 mg/d)	Higher intake (≥ 2.50 mg/d)
Total plant-based nitrite			
Model 1^a^ (n = 83,026)	Reference	0.814 (0.783–0.846) **	0.810 (0.765–0.857) **
Model 2^b^ (n = 83,026)	Reference	0.860 (0.827–0.895) **	0.860 (0.812–0.910) **
Model 3^c^ (n = 83,026)	Reference	0.866 (0.832–0.901) **	0.865 (0.817–0.917) **

	Lower intake (≤ 0.44 mg/d)	Moderate intake (0.45 -0.73 mg/d)	Higher intake (≥ 0.74 mg/d)
Total animal-based nitrite			
Model 1^a^ (n = 83,026)	Reference	0.927 (0.888–0.968) **	1.017 (0.974–1.061)
Model 2^b^ (n = 83,026)	Reference	0.954 (0.913–0.996) *	1.016 (0.973–1.061)
Model 3^c^ (n-83026)	Reference	0.954 (0.913–0.996) *	1.007 (0.964–1.052)

a; adjusted for age and sex, b; adjusted for age, sex, ethnicity, BMI, smoking, alcohol intake, educational level, socioeconomic status (Townsend deprivation index), physical activity level, c; adjusted for the covariates in Model 2 and intakes of (1) processed meat, red meat, poultry, fish, dairy, refined grains, coffee, tea, soft drinks, and sugar and confectionary when plant-sourced nitrate or nitrite were the exposures of interest, and (2) vegetables, fruits, wholegrains, refined grains, coffee, tea, soft drinks, and sugar and confectionary when animal-sourced nitrate or nitrite were the exposures of interest., *; *p* < 0.05, **; *p* < 0.001

### Associations between animal-sourced nitrate and nitrite and self-reported risk of periodontal disease

Animal-sourced nitrate was not associated with odds of periodontal disease in the primary cross-sectional or longitudinal analyses (Tables 2, 3). High but not moderate intakes of animal nitrite were associated with significantly higher odds of periodontal disease in cross-sectional analyses (OR [95%CI]: Moderate: 0.988 [0.956–1.020]; High: 1.043 [1.009–1.078]) compared to the low intake reference group (Tables 2). In longitudinal analyses, high intake of animal- sourced nitrite was not significantly associated with odds of periodontal disease, whereas moderate intake was associated with lower odds (OR [95%CI]: Moderate: 0.954 [0.913–0.996]; High: 1.007 [0.964–1.052]) compared to the low intake reference group (Tables 3).

### Subgroup and sensitivity analyses

**Fig. 1:**
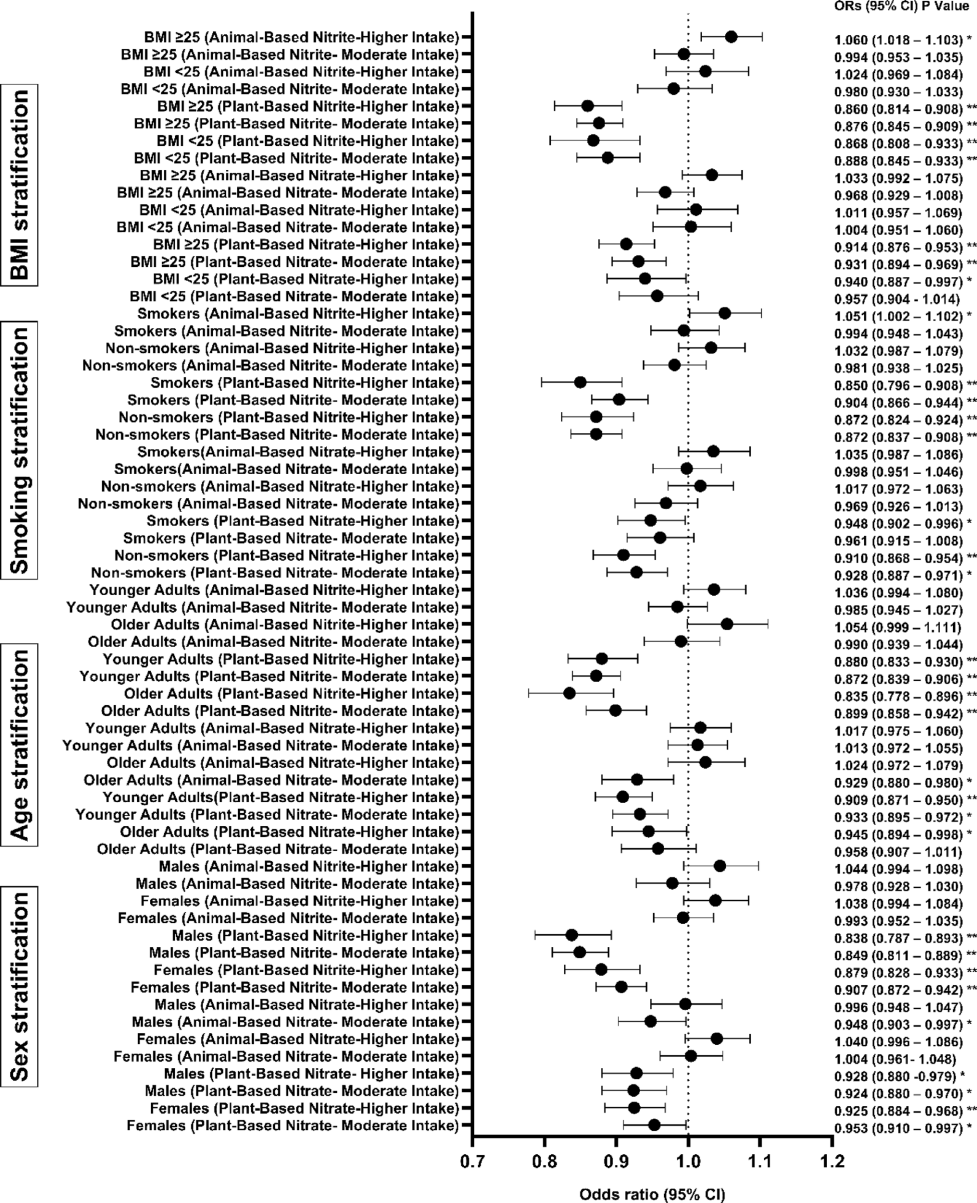
Cross-sectional analysis of the associations between nitrate/nitrite intake and odds (95% CI) of periodontal disease stratified by sex, age, smoking status, and body mass index in the UK Biobank study

In sub-group analyses, the cross-sectional associations between source-dependent nitrate and nitrite intake and odds of periodontal disease were similar across most population sub-groups (Fig. 1). The overall pattern of results was similar in sensitivity analyses for both cross-sectional and longitudinal analyses, particularly with regard to the association between plant-sourced nitrate/nitrite and the odds of periodontal disease (Supplementary Tables S2–S16). However, when intake was reclassified into quartiles (Supplementary Table S2) or when the analysis was restricted to participants who provided more than one dietary report (Supplementary Table S3) in the cross-sectional analysis, the association between animal-source nitrate intake and higher odds of periodontal disease was statistically significant. In the main longitudinal analysis, moderate nitrite intake from an animal source was associated with significantly lower odds of periodontal disease; however, this association was weakened and lost statistical significance when the sample was restricted to participants with at least two dietary reports, when dietary reports with implausible energy intake values were excluded, and when they were categorised into quartiles and quintiles and when including the Eatwell Guide score as a covariate (Supplementary Table S4–S8). When examining sub-sources of nitrate and nitrite, vegetable-derived nitrate was associated with lower odds of periodontal disease both cross-sectionally and longitudinally, while vegetable-derived nitrite was significantly associated with lower odds at moderate or high intakes in the longitudinal analysis and only at moderate intakes in the cross-sectional analysis. In contrast, nitrite from unprocessed meat was associated with slightly higher odds of periodontal disease in cross-sectional analyses only (Supplementary Tables S9, S10). Cross-sectional analyses excluding those participants who provided their oral measurements at the baseline visit before completing the Oxford WebQ questionnaire showed consistency regarding nitrate and plant-sourced nitrite. However, moderate intake of animal-sourced nitrite was associated with lower odds of periodontal disease (Supplementary Table S11). In individual oral health outcome cross-sectional and longitudinal analyses, plant nitrate and nitrite were associated with lower odds of bleeding gums, loose teeth, and denture use. In contrast, animal -sourced nitrate and nitrite were associated with higher bleeding gums at higher intakes in the cross-sectional analysis only, while the longitudinal analysis showed an association between higher intake of animal-sourced nitrate and higher gingival pain (Supplementary Tables S12, S13).

## Discussion

This study found that moderate and higher intakes of plant-derived nitrate and nitrite were consistently associated with lower odds of self-reported periodontal disease in middle-aged and older adults, both cross-sectionally and longitudinally. Evidence for animal-derived nitrate and nitrite was weaker and less consistent. Animal-derived nitrate was not associated with periodontal disease in the primary analyses, although weak positive associations were observed in some sensitivity analyses. Meanwhile, higher animal-derived nitrite was associated with higher odds of periodontal disease in the primary cross-sectional analysis, but this was not observed longitudinally. These findings suggest that the relationship between dietary nitrate/nitrite and periodontal disease may depend on food source, with more consistent evidence for potentially favourable associations with plant-derived sources.

Previous studies indicate that the associations between nitrate/nitrite and health outcomes vary by source. Specifically, plant-derived nitrate is associated with lower systolic and diastolic blood pressure, lower risk of cardiovascular disease [55], lower frailty [56], and lower all-cause mortality [32]. On the other hand, animal-derived nitrate and nitrite are detrimentally associated with mortality [32]. To our knowledge, the present study is the first to investigate associations between source-specific dietary nitrate and nitrite intake and oral health outcomes in a large cohort study. Our results align with the general trend observed in previous studies, supporting a protective role of plant-derived nitrate and nitrite against adverse health outcomes.

A potential mechanism through which plant-based nitrate impacts oral health is the modification of the oral microbiota, particularly by increasing the abundance of nitrate-reducing, health-promoting bacteria, such as *Rothia* and *Neisseria*. This has been demonstrated in several randomised controlled trials using vegetable-based nitrate-rich supplements [25, 57–60]. These bacteria are involved in pH buffering as part of the nitrate-reduction process, using lactate as an electron donor and carbon source and raising salivary pH, which could contribute towards a healthy oral environment [27]. Additional evidence comes from Rosier et al.[61], who reported that the proportion of nitrate-reducing bacteria was significantly lower in periodontitis patients than in healthy individuals across five countries (Japan, USA, Spain, Chile, Brazil), despite marked differences in ethnicity and dietary habits. One of the leading causes of periodontal disease is dysbiosis [62], with early stages characterised by distinct shifts in the oral microbiome [63]. These include a 21% reduction in *Rothia*, which is detrimentally correlated with the severity of gingival bleeding [63]. Rosier et al.[61] also reported that nitrate-reducing bacteria were inversely associated with periodontitis-associated taxa and full mouth plaque scores. Mechanistic evidence is further strengthened by microbial dysbiosis analyses. Specifically, a reanalysis of the data from Jockel-Schneider et al.[58] showed a significant decrease in dysbiosis among periodontal patients who consumed nitrate-rich lettuce juice, suggesting that plant-based nitrate promotes the restoration of oral microbial balance [64]. Beyond the microbiota, plant-based nitrate also increases the production of nitric oxide, which has antimicrobial properties [65] and increase blood flow to oral tissues [66]. While plant-based nitrite could increase nitric oxide production similarly as nitrate, it would not serve as a substrate for nitrate-reducing bacteria, so it is not clear why the association with oral health is so apparent.

Animal-derived nitrate and nitrite showed more complex and less robust associations. No significant association was found between animal-derived nitrate and periodontal disease in primary analyses, although some sensitivity analyses suggested weak positive associations at higher intakes. Meanwhile, higher animal-derived nitrite was associated with higher odds of periodontal disease in the primary cross-sectional analysis only, with no adverse association observed longitudinally. One possible explanation for an association between high animal nitrite and odds of periodontal disease is that animal-foods often contain compounds such as amines, amides, and heme iron, which can promote nitrosative chemistry and oxidative stress in the presence of nitrite, particularly when antioxidants availability (e.g., vitamin C, vitamin E, and polyphenols) is low [67, 68]. A recent review by Rosier et al.[69] highlights that the metabolism of nitrate and nitrite can follow two opposing pathways. When nitrate is derived from plant sources rich in antioxidants, it’s reduction—initiated by the oral microbiome—favours production of nitric oxide, which has protective properties and promotes microbial balance [69]. Conversely, in environments rich in oxidising agents or heme iron, nitrite may be preferentially converted into N-nitrosamines and reactive nitrogen species, which exacerbate oxidative stress and reduce nitric oxide bioavailability [69]. Such reactions could occur locally in the mouth, where nitrite is actively metabolised by the oral bacteria, potentially leading to damage to oral tissues, microbial dysbiosis and periodontal inflammation when animal sourced nitrite is consumed [70]. However, current evidence is insufficient to establish a direct causal relationship—especially given the association between animal nitrite and increased odds of periodontal disease was only observed cross-sectionally in the present study and associations were not consistent across all sensitivity analyses—such that this remains a hypothesis at present, and future mechanistic research is needed to evaluate the effects of different nitrite sources on oral nitric oxide availability and periodontal health.

In the cross-sectional analysis of nitrite sub-sources derived from unprocessed and processed meats, we found a significant positive association between higher intake of unprocessed meat-derived nitrite and the risk of periodontal disease. Unprocessed meat sources of nitrate and nitrite (e.g. red meat and fish) typically contribute greater absolute amounts of these compounds to intake than processed meats, which have nitrate and nitrite added as preservatives [40], and it is possible that higher absolute amounts nitrite are required to detect appreciable effects on oral health.

### Strengths and limitations

The present study has several strengths. Firstly, our study included a large sample of 158,778 participants in cross-sectional analyses and 83,026 participants in longitudinal analyses, which ensured statistical power for our analyses. Additionally, our inclusion of longitudinal analyses reduced the likelihood of reverse causality (i.e., poor oral health influencing food intake). Additionally, we conducted an extensive set of sensitivity analyses and sub-analyses, and the associations remained reasonably robust. Nevertheless, some limitations should be highlighted. Firstly, nitrate and nitrite intake was estimated based on a comprehensive food database that includes common foods in the UK. As the nitrate and nitrite content can vary within the same foods based on cultivation practices, cooking and environmental conditions for storage, there is potential for measurement error when estimating intake. In addition, the estimated plant-based nitrite intake was markedly higher than animal-based nitrite intake, which is unexpected [71] and could partly reflect methodological factors related to the quantification of these anions in food composition studies. In particular, commonly used analytical methods such as the Griess reaction can be susceptible to interference from plant-derived compounds, including anthocyanins, potentially leading to overestimation of nitrite concentrations in plant-based foods if not adequately controlled for [72]. Secondly, participants identified as ‘at risk’ for periodontal disease in this study were based on self-reported questionnaires of oral health symptoms, including bleeding gums, gum pain, or loose teeth, without the use of clinical diagnostic tools. This could increase the risk of misclassification error. However, previous studies have indicated that these symptoms are valid proxies for clinician-diagnosed periodontal disease risk [42, 43]. Third, attributing the observed lower risk of periodontal disease specifically to plant-derived nitrate/nitrite is challenging because plant sources also contain various other nutrients, such as dietary antioxidants, that are associated with mitigating periodontal disease [73–76]. However, we saw a similar pattern of association in our analyses even when controlling for diet quality. This suggests that our results may not be due to confounding related to the wider diet of participants. Fourth, due to the observational nature of this study, it was not possible to infer causality. Fifth, the UK Biobank study did not collect data on oral hygiene practices, such as toothbrushing frequency, brushing duration, and the use of mouthwash or dental floss, which impact oral health. Therefore, we were unable to control for these parameters in our analyses. Sixth, all covariates used in the longitudinal analysis, such as BMI, smoking status, and physical activity, were taken from the baseline assessment visit, but may have changed during the follow-up period. Therefore, confounding due to changes in these factors over time cannot be excluded. Finally, the majority of participants in the UK Biobank study are of White ethnicity and have a healthier lifestyle and a higher standard of living compared to the UK population [47]. Whilst comparisons with representative population studies suggest that this volunteer selection bias does not have a major impact on the ability to accurately identify risk factor associations [77], further studies across diverse populations using alternative oral health assessment tools may still be valuable to test the consistency of the findings, enhance their generalisability and providing more comprehensive and in-depth insights.

## Conclusion

The results of this study indicate a divergent association between dietary nitrate and nitrite sources and the odds of periodontal disease. Plant-derived nitrate and nitrite were associated with lower odds of periodontal disease in this population, while higher intake of animal-derived nitrite was associated with higher odds in the primary cross-sectional but not longitudinal analyses, and these findings were not robust across all sensitivity analyses. Increasing intake of plant foods rich in nitrate could represent a strategy to improve oral health and lower periodontal disease odds – something which requires further investigation in large-scale randomised controlled trials. In this context, the results reinforce existing public health recommendations to consume plant/vegetable-rich diets and indicate that such dietary patterns may support oral health as well as established cardiometabolic benefits.

## Supplementary Information

Below is the link to the electronic supplementary material.

Supplementary Material 1

## Data Availability

The data used in this study are available from the UK Biobank upon application and approval (www.ukbiobank.ac.uk).
