# Supplementary material for: Exploring the associations between dietary nitrate and nitrite intake and markers of self-reported periodontal disease in the UK Biobank cohort
Source: Eur J Nutr. 2026 Jun 29;65(5):174. doi: 10.1007/s00394-026-04035-2 (PMC13315416; doi:10.1007/s00394-026-04035-2)
Supplement: Supplementary file 1 — Supplementary Material 1 [file 394_2026_4035_MOESM1_ESM.docx]

**Table S1.** Classification of Nitrate and Nitrite intake into 12 food groups, derived from Oxford WebQ dietary data collected in the UK Biobank study.

| **Food Group** | **Included** | **Units** |
| --- | --- | --- |
| 1. Green leafy vegetables | Including lettuce, kale, spinach, chard, etc. | mg/day |
| 2. Fruit | Including juices, dried and whole fruits. | mg/day |
| 3. Potatoes | Including only white variety [boiled, mashed, baked or in soup/recipes excluding deep fried or packaged potato chips/crips]. | mg/day |
| 4. Other vegetables | Including cruciferous, allium and root vegetables [excluding white potato] as well as beans, legumes and fruits traditionally consumed as vegetables [e.g., tomato] whether raw, cooked or as juice. | mg/day |
| 5. Other plant sources | Including nuts, seeds, oils, tea, coffee, tofu, herbs and spices. | mg/day |
| 6. Meat and fish | Including red meats, poultry, and seafoods. | mg/day |
| 7. Processed meats | Including ham, salami and bacon. | mg/day |
| 8. Other animal sauces | Including dairy [milk, butter, yoghurt, cheese, etc], eggs or lard. | mg/day |
| 9. Wholegrains | Including only wholemeal/grain breads or pastas, brown rice, or whole food sources [e.g., oats, barley, shredded wheat, etc]. | mg/day |
| 10. Alcohol | Including all beer, wine and liquors. | mg/day |
| 11. Discretionary and miscellaneous | Including confectionary, cakes, savory snacks, refined grains [e.g., white rice, pasta, or bread and processed cereals], fried chips and crisps, soft drinks, cordial, ice cream, condiments [e.g., mayonnaise, ketchup etc.,], honey, jam, chocolate, and mixed spreadable fats. | mg/day |
| 12. Water | Including glasses drunk daily [tap or bottled]. | mg/day |


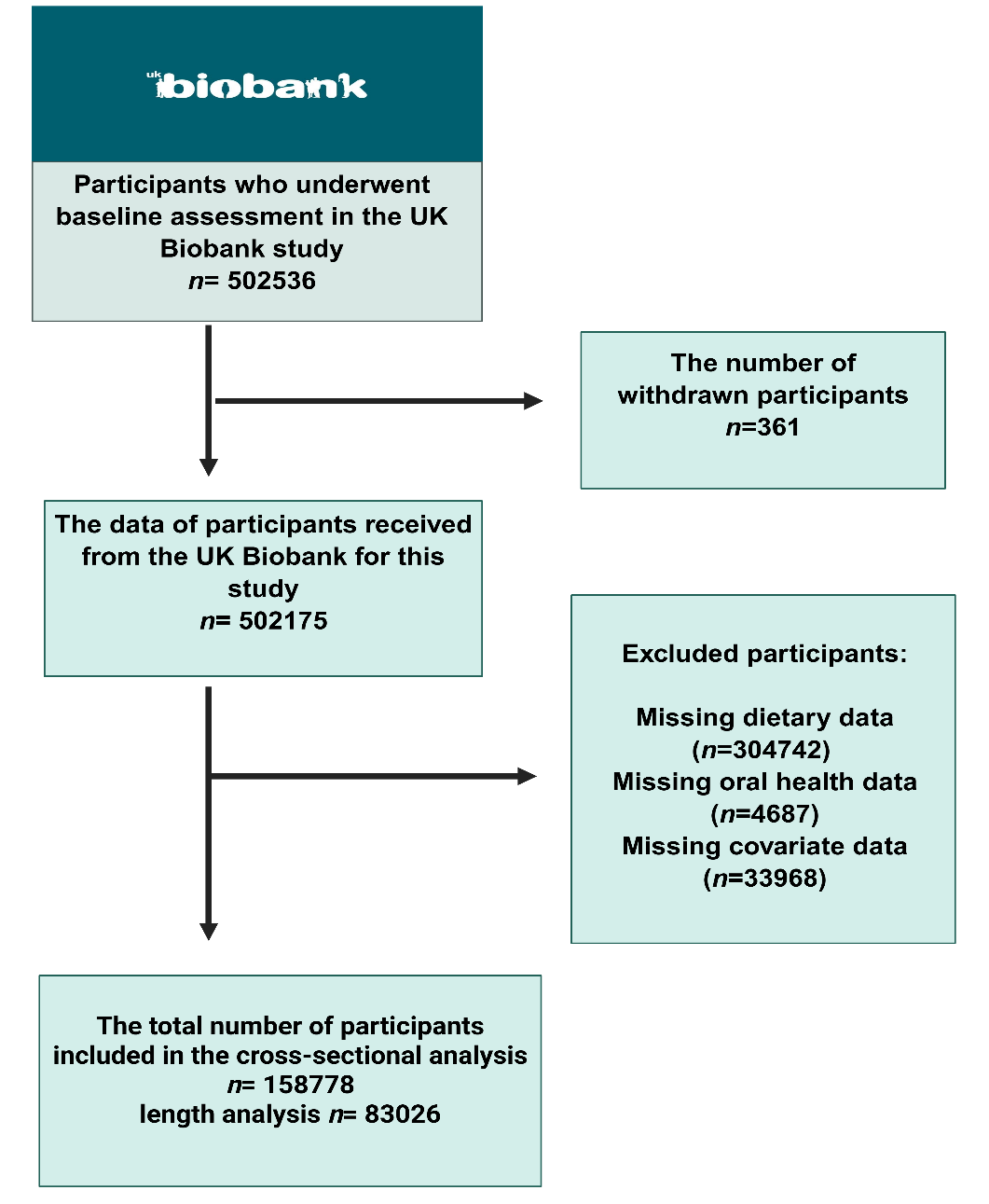


**Figure S1.** Flow chart showing participants undergoing baseline assessment in the UK Biobank study and the final data set included in the analysis.

**Table S2.** Fully adjusted cross-sectional analysis of the nitrate/nitrite intakes and Odds (95% CI) of periodontal disease in the UK Biobank study when recategorising nitrate/ nitrite intakes into quartiles (n=158778).

| Very lower Intake | Lower Intake | Moderate Intake | Higher Intake |
| --- | --- | --- | --- |
| Total Plant-Based Nitrate | | | |
| Reference | 0.951 (0.916 – 0.988) * | 0.905 (0.871 – 0.941) ** | 0.915 (0.880 – 0.952) ** |
| Total Animal-Based Nitrate | | | |
| Reference | 0.989 (0.953 – 1.027) | 0.986 (0.950 – 1.024) | 1.039 (1.001 – 1.079) * |
| Total Plant-Based Nitrite | | | |
| Reference | 0.908 (0.880 – 0.937) ** | 0.848 (0.817 – 0.880) ** | 0.855 (0.809 – 0.903) ** |
| Total Animal-Based Nitrite | | | |
| Reference | 0.970 (0.934 – 1.007) | 1.006 (0.969 – 1.044) | 1.042 (1.004 – 1.082) * |

*; p < 0.05, **; p < 0.001.

**Table S3.** Fully adjusted cross-sectional analysis of the nitrate/nitrite intakes and Odds (95% CI) of periodontal disease in the UK Biobank study when including two or more dietary reports (n=103084).

| Total Plant-Based Nitrate | | | |
| --- | --- | --- | --- |
|  | **Lower Intake** | **Moderate Intake** | **Higher Intake** |
| Model 3^c^ | Reference | 0.932 (0.894 – 0.971) ** | 0.933 (0.894 – 0.974) * |
| Total Animal-Based Nitrate | | | |
|  | **Lower Intake** | **Moderate Intake** | **Higher Intake** |
| Model 3^c^ | Reference | 1.012 (0.971 – 1.054) | 1.055 (1.012 – 1.098) * |
| Total Plant-Based Nitrite | | | |
|  | **Lower Intake** | **Moderate Intake** | **Higher Intake** |
| Model 3^c^ | Reference | 0.874 (0.842 – 0.908) ** | 0.849 (0.803 – 0.898) ** |
| Total Animal-Based Nitrite | | | |
|  | **Lower Intake** | **Moderate Intake** | **Higher Intake** |
| Model 3^c^ | Reference | 1.003 (0.963 – 1.045) | 1.063 (1.020 – 1.107) * |

c; adjusted for the covariates in Model 2 and intakes of (a) processed meat, red meat, poultry, fish, dairy, refined grains, coffee, tea, soft drinks, and sugar and confectionary when plant-sourced nitrate or nitrite were the exposures of interest, and (b) vegetables, fruits, wholegrains, refined grains, coffee, tea, soft drinks, and sugar and confectionary when animal-sourced nitrate or nitrite were the exposures of interest., *; p < 0.05, **; p < 0.001.

**Table S4.** Fully adjusted longitudinal analysis of the nitrate/nitrite intakes and Odds (95% CI) of periodontal disease in the UK Biobank study when including two or more dietary reports (n=52706).

| Total Plant-Based Nitrate | | | |
| --- | --- | --- | --- |
|  | **Lower Intake** | **Moderate Intake** | **Higher Intake** |
| Model 3^c^ | Reference | 0.873 (0.826 – 0.924) ** | 0.895 (0.844 – 0.948) ** |
| Total Animal-Based Nitrate | | | |
|  | **Lower Intake** | **Moderate Intake** | **Higher Intake** |
| Model 3^c^ | Reference | 1.002 (0.948 – 1.059) | 1.052 (0.995 – 1.111) |
| Total Plant-Based Nitrite | | | |
|  | **Lower Intake** | **Moderate Intake** | **Higher Intake** |
| Model 3^c^ | Reference | 0.867 (0.825 – 0.912) ** | 0.841 (0.780 – 0.908) ** |
| Total Animal-Based Nitrite | | | |
|  | **Lower Intake** | **Moderate Intake** | **Higher Intake** |
| Model 3^c^ | Reference | 0.973 (0.921 – 1.028) | 1.011 (0.956 – 1.068) |

c; adjusted for the covariates in Model 2 and intakes of (a) processed meat, red meat, poultry, fish, dairy, refined grains, coffee, tea, soft drinks, and sugar and confectionary when plant-sourced nitrate or nitrite were the exposures of interest, and (b) vegetables, fruits, wholegrains, refined grains, coffee, tea, soft drinks, and sugar and confectionary when animal-sourced nitrate or nitrite were the exposures of interest., *; p < 0.05, **; p < 0.001.

**Table S5.** Fully adjusted longitudinal analysis of the nitrate/ nitrite intakes, excluding dietary reports with implausible energy intake values and Odds (95% CI) of periodontal disease in the UK Biobank study (n=50833).

| Total Plant-Based Nitrate | | | |
| --- | --- | --- | --- |
|  | **Lower Intake** | **Moderate Intake** | **Higher Intake** |
| Model 3^c^ | Reference | 0.880 (0.833 – 0.930) ** | 0.880 (0.831 – 0.932) ** |
| Total Animal-Based Nitrate | | | |
|  | **Lower Intake** | **Moderate Intake** | **Higher Intake** |
| Model 3^c^ | Reference | 0.986 (0.934 – 1.042) | 1.029 (0.974 – 1.087) |
| Total Plant-Based Nitrite | | | |
|  | **Lower Intake** | **Moderate Intake** | **Higher Intake** |
| Model 3^c^ | Reference | 0.863 (0.822 – 0.906) ** | 0.863 (0.803 – 0.927) ** |
| Total Animal-Based Nitrite | | | |
|  | **Lower Intake** | **Moderate Intake** | **Higher Intake** |
| Model 3^c^ | Reference | 0.947 (0.897 – 1.000) | 1.004 (0.951 – 1.060) |

c; adjusted for the covariates in Model 2 and intakes of (a) processed meat, red meat, poultry, fish, dairy, refined grains, coffee, tea, soft drinks, and sugar and confectionary when plant-sourced nitrate or nitrite were the exposures of interest, and (b) vegetables, fruits, wholegrains, refined grains, coffee, tea, soft drinks, and sugar and confectionary when animal-sourced nitrate or nitrite were the exposures of interest., *; p < 0.05, **; p < 0.001.

**Table S6.** Fully adjusted longitudinal analysis of the nitrate/ nitrite intakes and Odds (95% CI) of periodontal disease in the UK Biobank study when recategorising nitrate/ nitrite intakes into quartiles (n=83026).

| Very lower Intake | Lower Intake | Moderate Intake | Higher Intake |
| --- | --- | --- | --- |
| Total Plant-Based Nitrate | | | |
| Reference | 0.883 (0.840 – 0.929) ** | 0.843 (0.800 – 0.887) ** | 0.853 (0.809 – 0.899) ** |
| Total Animal-Based Nitrate | | | |
| Reference | 0.990 (0.942 -1.041) | 0.959 (0.912 – 1.009) | 1.053 (1.001 – 1.107) * |
| Total Plant-Based Nitrite | | | |
| Reference | 0.878 (0.842 – 0.916) ** | 0.824 (0.784 -0.866) ** | 0.867 (0.806 – 0.932) ** |
| Total Animal-Based Nitrite | | | |
| Reference | 0.972 (0.925 – 1.022) | 0.986 (0.938 – 1.036) | 1.009 (0.960 – 1.061) |

*; p < 0.05, **; p < 0.001.

**Table S7.** Fully adjusted longitudinal analysis of the nitrate/ nitrite intakes and Odds (95% CI) of periodontal disease in the UK Biobank study when recategorising nitrate/ nitrite intakes into quintiles (n=83026).

| Very lower Intake | Lower Intake | Moderate Intake | Higher Intake | Very Higher Intake |
| --- | --- | --- | --- | --- |
| Total Plant-Based Nitrate | | | | |
| Reference | 0.911 (0.861 – 0.963) ** | 0.835 (0.789 – 0.884) ** | 0.839 (0.739 – 0.889) ** | 0.849 (0.801 – 0.900) ** |
| Total Animal-Based Nitrate | | | | |
| Reference | 0.990 (0.936 – 1.047) | 0.972 (0.919 – 1.039) | 0.982 (0.928 – 1.039) | 1.057 (0.999 – 1.117) |
| Total Plant-Based Nitrite | | | | |
| Reference | 0.846 (0.808 – 0.885) ** | 0.803 (0.763 – 0.844) ** | 0.787 (0.741 – 0.836) ** | 0.887 (0.815 – 0.966) * |
| Total Animal-Based Nitrite | | | | |
| Reference | 0.979 (0.927 – 1.035) | 0.945 (0.894 – 0.999) * | 1.007 (0.952 – 1.064) | 1.006 (0.951 – 1.064) |

*; p < 0.05, **; p < 0.001.

**Table S8.** Fully adjusted longitudinal analysis of the nitrate/ nitrite intakes and Odds (95% CI) of periodontal disease in the UK Biobank study when including the Eatwell Guide score as a covariate (n=83026).

| Total Plant-Based Nitrate | | | |
| --- | --- | --- | --- |
|  | **Lower Intake** | **Moderate Intake** | **Higher Intake** |
| Model 3^c^ | Reference | 0.908 (0.868 – 0.950) ** | 0.921 (0.878 – 0.966) ** |
| Total Animal-Based Nitrate | | | |
|  | **Lower Intake** | **Moderate Intake** | **Higher Intake** |
| Model 3^c^ | Reference | 0.987 (0.945 – 1.032) | 1.025 (0.981 – 1.071) |
| Total Plant-Based Nitrite | | | |
|  | **Lower Intake** | **Moderate Intake** | **Higher Intake** |
| Model 3^c^ | Reference | 0.889 (0.853 – 0.925) ** | 0.892 (0.841 – 0.946) ** |
| Total Animal-Based Nitrite | | | |
| (mg/d) | **Lower Intake** | **Moderate Intake** | **Higher Intake** |
| Model 3^c^ | Reference | 0.961 (0.920 – 1.003) | 1.001 (0.958 – 1.045) |

c; adjusted for the covariates in Model 2 and intakes of (a) processed meat, red meat, poultry, fish, dairy, refined grains, coffee, tea, soft drinks, and sugar and confectionary when plant-sourced nitrate or nitrite were the exposures of interest, and (b) vegetables, fruits, wholegrains, refined grains, coffee, tea, soft drinks, and sugar and confectionary when animal-sourced nitrate or nitrite were the exposures of interest., *; p < 0.05, **; p < 0.001.

**Table S9.** Fully adjusted cross-sectional analysis of the associations between sub-groups of nitrate/nitrite sources and Odds (95% CI) of periodontal disease in the UK Biobank study (n=158778).

| Vegetable-Derived Nitrate ^a^ | | | |
| --- | --- | --- | --- |
|  | **Lower Intake (≤ 25.23)** | **Moderate Intake (25.24 – 66.57)** | **Higher Intake (≥ 66.58)** |
| Model 3^c^ | Reference | 0.957 (0.927 – 0.989) * | 0.936 (0.905 – 0.968) ** |
| Unprocessed Meat Sources of Nitrate ^b^ | | | |
|  | **Lower Intake (≤ 2.06)** | **Moderate Intake (2.07 – 3.99)** | **Higher Intake (≥ 4)** |
| Model 3^c^ | Reference | 0.975 (0.944 – 1.007) | 1.023 (0.990 – 1.057) |
| Processed Meat Sources of Nitrate | | | |
|  | **Lower Intake (≤ 0.00)** | **Moderate Intake (0.01 - .008)** | **Higher Intake (≥ 0.09)** |
| Model 3^c^ | Reference | 0.953 (0.897 – 1.014) | 0.989 (0.962 – 1.018) |
| Vegetable-Derived Nitrite ^a^ | | | |
| (mg/d) | **Lower Intake (≤ 0.16)** | **Moderate Intake (0.17-0.31)** | **Higher Intake (≥ 0.32)** |
| Model 3^c^ | Reference | 0.963 (0.932 – 0.994) * | 0.984 (0.952 – 1.018) |
| Unprocessed Meat Sources of Nitrite^b^ | | | |
|  | **Lower Intake (≤ 0.36)** | **Moderate Intake (0.37 – 0.58)** | **Higher Intake (≥ 0.59)** |
| Model 3^c^ | Reference | 1.007 (0.975 – 1.040) | 1.038 (1.005 – 1.072) * |
| Processed Meat Sources of Nitrite | | | |
|  | **Lower Intake (≤ 0.00)** | **Moderate Intake (0.01 – 0.07)** | **Higher Intake (≥ 0.8)** |
| Model 3^c^ | Reference | 0.941 (0.886 – 1.000) | 0.992 (0.964 – 1.021) |

a; Potatoes, green-leafy vegetables, other vegetables, b; Meats, fish and other animal sources, c; adjusted for the covariates in Model 2 and intakes of (a) processed meat, red meat, poultry, fish, dairy, refined grains, coffee, tea, soft drinks, and sugar and confectionary when plant-sourced nitrate or nitrite were the exposures of interest, and (b) vegetables, fruits, wholegrains, refined grains, coffee, tea, soft drinks, and sugar and confectionary when animal-sourced nitrate or nitrite were the exposures of interest., *; p < 0.05, **; p < 0.001.

**Table S10.** Fully adjusted longitudinal analysis of the associations between sub-groups of nitrate/nitrite sources and Odds (95% CI) of periodontal disease in the UK Biobank study (n=83026).

| Vegetable-Derived Nitrate ^a^ | | | |
| --- | --- | --- | --- |
|  | **Lower Intake (≤ 25.23)** | **Moderate Intake (25.24 – 66.57)** | **Higher Intake (≥ 66.58)** |
| Model 3^c^ | Reference | 0.894 (0.856 – 0.934) ** | 0.887 (0.848 – 0.928) ** |
| Unprocessed Meat Sources of Nitrate^b^ | | | |
|  | **Lower Intake (≤ 2.06)** | **Moderate Intake (2.07 – 3.99)** | **Higher Intake (≥ 4)** |
| Model 3^c^ | Reference | 0.982 (0.940 – 1.026) | 1.037 (0.993 – 1.084) |
| Processed Meat Sources of Nitrate | | | |
|  | **Lower Intake (≤ 0.00)** | **Moderate Intake (0.01 - .008)** | **Higher Intake (≥ 0.09)** |
| Model 3^c^ | Reference | 0.963 (0.889 – 1.044) | 0.982 (0.945 – 1.020) |
| Vegetable-Derived Nitrite ^a^ | | | |
| (mg/d) | **Lower Intake (≤ 0.16)** | **Moderate Intake (0.17-0.31)** | **Higher Intake (≥ 0.32)** |
| Model 3^c^ | Reference | 0.919 (0.881 – 0.960) ** | 0.947 (0.905 – 0.990) * |
| Unprocessed Meat Sources of Nitrite ^b^ | | | |
|  | **Lower Intake (≤ 0.36)** | **Moderate Intake (0.37 – 0.58)** | **Higher Intake (≥ 0.59)** |
| Model 3^c^ | Reference | 0.972 (0.931 – 1.015) | 1.016 (0.973 – 1.061) |
| Processed Meat Sources of Nitrite | | | |
|  | **Lower Intake (≤ 0.00)** | **Moderate Intake (0.01 – 0.07)** | **Higher Intake (≥ 0.8)** |
| Model 3^c^ | Reference | 0.947 (0.875 – 1.025) | 0.985 (0.947 – 1.023) |

a; Potatoes, green-leafy vegetables, other vegetables, b; Meats, fish and other animal sources, c; adjusted for the covariates in Model 2 and intakes of (a) processed meat, red meat, poultry, fish, dairy, refined grains, coffee, tea, soft drinks, and sugar and confectionary when plant-sourced nitrate or nitrite were the exposures of interest, and (b) vegetables, fruits, wholegrains, refined grains, coffee, tea, soft drinks, and sugar and confectionary when animal-sourced nitrate or nitrite were the exposures of interest., *; p < 0.05, **; p < 0.001.

**Table S11.** Fully adjusted cross-sectional analysis of nitrate/nitrite intake and odds ratio (95% CI) for periodontal disease in the UK Biobank study when excluding oral measurements taken at the baseline visit prior to completion of the Oxford WebQ questionnaire (n = 51945).

| Total Plant-Based Nitrate | | | |
| --- | --- | --- | --- |
|  | **Lower Intake** | **Moderate Intake** | **Higher Intake** |
| Model 3^c^ | Reference | 0.876 (0.827 – 0.929) ** | 0.868 (0.820 – 0.918) ** |
| Total Animal-Based Nitrate | | | |
|  | **Lower Intake** | **Moderate Intake** | **Higher Intake** |
| Model 3^c^ | Reference | 0.953 (0.900 – 1.008) | 0.975 (0.922 – 1.031) |
| Total Plant-Based Nitrite | | | |
|  | **Lower Intake** | **Moderate Intake** | **Higher Intake** |
| Model 3^c^ | Reference | 0.869 (0.808 – 0.935) ** | 0.860 (0.818 – 0.906) ** |
| Total Animal-Based Nitrite | | | |
| (mg/d) | **Lower Intake** | **Moderate Intake** | **Higher Intake** |
| Model 3^c^ | Reference | 0.936 (0.883 – 0.991) * | 0.992 (0.939 – 1.049) |

c; adjusted for the covariates in Model 2 and intakes of (a) processed meat, red meat, poultry, fish, dairy, refined grains, coffee, tea, soft drinks, and sugar and confectionary when plant-sourced nitrate or nitrite were the exposures of interest, and (b) vegetables, fruits, wholegrains, refined grains, coffee, tea, soft drinks, and sugar and confectionary when animal-sourced nitrate or nitrite were the exposures of interest., *; p < 0.05, **; p < 0.001.

**Table S12.** Fully adjusted cross-sectional analysis of the nitrate/nitrite intakes and Odds (95% CI) of individual oral health outcomes in the UK Biobank study (n=158778).

| Individual oral health outcomes | Lower Intake | Moderate Intake | Higher Intake |
| --- | --- | --- | --- |
| Total Plant-Based Nitrate | | | |
| Painful gums | Reference | 0.967 (0.895 -1.045) | 0.972 (0.898 – 1.052) |
| Bleeding gums | Reference | 0.936 (0.903 – 0.971) ** | 0.924 (0.890 – 0.960) ** |
| Loose teeth | Reference | 0.920 (0.862 – 0.983) * | 0.907 (0.847 – 0.971) * |
| Dentures | Reference | 0.951 (0.913 – 0.989) * | 0.904 (0.867 – 0.942) ** |
| Toothache | Reference | 0.956 (0.899 – 1.017) | 0.981 (0.920 – 1.046) |
| Mouth ulcers | Reference | 0.985 (0.945 – 1.026) | 1.034 (0.992 – 1.078) |
| Total Animal-Based Nitrate | | | |
| Painful gums | Reference | 1.007 (0.934 – 1.087) | 1.003 (0.929 – 1.082) |
| Bleeding gums | Reference | 0.997 (0.961 – 1.033) | 1.044 (1.007 – 1.082) * |
| Loose teeth | Reference | 0.917 (0.859 – 0.979) * | 1.003 (0.940 – 1.069) |
| Dentures | Reference | 1.003 (0.964 – 1.044) | 1.031 (0.991 – 1.073) |
| Toothache | Reference | 0.968 (0.910 – 1.029) | 0.979 (0.921 – 1.041) |
| Mouth ulcers | Reference | 0.994 (0.955 – 1.035) | 1.024 (0.983 – 1.065) |
| Total Plant-Based Nitrite | | | |
| Painful gums | Reference | 0.953 (0.889 – 1.021) | 1.023 (0.927 – 1.128) |
| Bleeding gums | Reference | 0.881 (0.853 – 0.911) ** | 0.832 (0.792 – 0.873) ** |
| Loose teeth | Reference | 0.846 (0.797 – 0.899) ** | 0.874 (0.801 – 0.954) * |
| Dentures | Reference | 0.921 (0.888 – 0.954) ** | 0.901 (0.855 – 0.950) ** |
| Toothache | Reference | 0.998 (0.944 – 1.055) | 1.036 (0.958 – 1.121) |
| Mouth ulcers | Reference | 1.019 (0.984 – 1.057) | 0.994 (0.943 – 1.047) |
| Total Animal-Based Nitrite | | | |
| Painful gums | Reference | 1.027 (0.953 – 1.107) | 1.022 (0.947 – 1.104) |
| Bleeding gums | Reference | 0.982 (0.947 – 1.018) | 1.055 (1.018 – 1.094) * |
| Loose teeth | Reference | 0.993 (0.930 – 1.061) | 1.057 (0.990 – 1.128) |
| Dentures | Reference | 1.022 (0.982 – 1.063) | 1.017 (0.977 – 1.059) |
| Toothache | Reference | 0.989 (0.930 – 1.052) | 0.986 (0.927 – 1.048) |
| Mouth ulcers | Reference | 0.983 (0.944 – 1.022) | 1.023 (0.983 – 1.065) |

*; p < 0.05, **; p < 0.001.

**Table S13.** Fully adjusted longitudinal analysis of the nitrate/nitrite intakes and Odds (95% CI) of individual oral health outcomes in the UK Biobank study (n=83026).

| Individual oral health outcomes | Lower Intake | Moderate Intake | Higher Intake |
| --- | --- | --- | --- |
| Total Plant-Based Nitrate | | | |
| Painful gums | Reference | 0.929 (0.838 – 1.029) | 0.997 (0.898 – 1.106) |
| Bleeding gums | Reference | 0.878 (0.835 -0.922) ** | 0.889 (0.845 – 0.935) ** |
| Loose teeth | Reference | 0.856 (0.786 – 0.931) ** | 0.875 (0.801 – 0.955) * |
| Dentures | Reference | 0.928 (0.892 – 0.966) ** | 0.916 (0.879 – 0.954) ** |
| Toothache | Reference | 0.965 (0.891 – 1.046) | 0.955 (0.878 – 1.039) |
| Mouth ulcers | Reference | 0.989 (0.933 – 1.043) | 1.000 (0.943 – 1.059) |
| Total Animal-Based Nitrate | | | |
| Painful gums | Reference | 0.983 (0.889 – 1.087) | 1.104 (1.001 – 1.219) * |
| Bleeding gums | Reference | 0.985 (0.939 – 1.034) | 1.021 (0.973 – 1.072) |
| Loose teeth | Reference | 0.977 (0.898 – 1.063) | 1.040 (0.957 – 1.131) |
| Dentures | Reference | 0.969 (0.931 – 1.008) | 0.986 (0.948 – 1.026) |
| Toothache | Reference | 1.041 (0.962 – 1.127) | 0.974 (0.898 – 1.056) |
| Mouth ulcers | Reference | 0.984 (0.932 – 1.039) | 0.982 (0.930 – 1.038) |
| Total Plant-Based Nitrite | | | |
| Painful gums | Reference | 0.910 (0.830 – 0.997) * | 1.013 (0.892 – 1.150) |
| Bleeding gums | Reference | 0.867 (0.830 – 0.907) ** | 0.848 (0.795 – 0.904) ** |
| Loose teeth | Reference | 0.873 (0.808 – 0.943) ** | 0.889 (0.794 – 0.994) * |
| Dentures | Reference | 0.939 (0.905 – 0.973) ** | 0.945 (0.898 – 0.996) * |
| Toothache | Reference | 0.977 (0.908 – 1.052) | 1.053 (0.952 – 1.166) |
| Mouth ulcers | Reference | 1.004 (0.955 – 1.054) | 1.011 (0.942 – 1.084) |
| Total Animal-Based Nitrite | | | |
| Painful gums | Reference | 0.928 (0.841 – 1.025) | 0.993 (0.899 – 1.096) |
| Bleeding gums | Reference | 0.961 (0.916 – 1.009) | 1.020 (0.972 – 1.071) |
| Loose teeth | Reference | 0.942 (0.866 – 1.025) | 0.978 (0.900 – 1.064) |
| Dentures | Reference | 0.950 (0.913 – 0.988) * | 0.963 (0.925 – 1.002) |
| Toothache | Reference | 1.006 (0.929 – 1.090) | 0.969 (0.894 – 1.050) |
| Mouth ulcers | Reference | 0.961 (0.911 – 1.015) | 0.986 (0.934 – 1.042) |

*; p < 0.05, **; p < 0.001.

**Table S14.** Fully adjusted cross-sectional analysis of the nitrate/nitrite intakes and Odds (95% CI) of periodontal disease in the UK Biobank study when recategorising nitrate/ nitrite intakes into quintiles (n=158778).

| Very lower Intake | Lower Intake | Moderate Intake | Higher Intake | Very Higher Intake |
| --- | --- | --- | --- | --- |
| Total Plant-Based Nitrate | | | | |
| Reference | 0.944 (0.905 – 0.984) * | 0.921 (0.883 – 0.961) ** | 0.902 (0.864 – 0.942) ** | 0.903 (0.865 – 0.944) ** |
| Total Animal-Based Nitrate | | | | |
| Reference | 0.983 (0.943 – 1.025) | 0.978 (0.938 – 1.020) | 0.992 (0.951 – 1.035) | 1.041 (0.999 – 1.086) |
| Total Plant-Based Nitrite | | | | |
| Reference | 0.894 (0.864 – 0.924) ** | 0.840 (0.809 – 0.872) | 0.824 (0.788 – 0.861) ** | 0.863 (0.809 – 0.921) ** |
| Total Animal-Based Nitrite | | | | |
| Reference | 0.970 (0.931 – 1.011) | 0.976 (0.937 – 1.018) | 1.013 (0.972 – 1.057) | 1.036 (0.994 – 1.080) |

*; p < 0.05, **; p < 0.001.

**Table S15.** Fully adjusted cross-sectional analysis of the nitrate/nitrite intakes, excluding dietary reports with implausible energy intake values and Odds (95% CI) of periodontal disease in the UK Biobank study (n=50833).

| Total Plant-Based Nitrate | | | |
| --- | --- | --- | --- |
|  | **Lower Intake** | **Moderate Intake** | **Higher Intake** |
| Model 3^c^ | Reference | 0.883 (0.834 – 0.936) ** | 0.881 (0.830 – 0.935) ** |
| Total Animal-Based Nitrate | | | |
|  | **Lower Intake** | **Moderate Intake** | **Higher Intake** |
| Model 3^c^ | Reference | 0.988 (0.933 – 1.045) | 1.027 (0.970 – 1.086) |
| Total Plant-Based Nitrite | | | |
|  | **Lower Intake** | **Moderate Intake** | **Higher Intake** |
| Model 3^c^ | Reference | 0.866 (0.823 – 0.912) ** | 0.864 (0.801 – 0.931) ** |
| Total Animal-Based Nitrite | | | |
|  | **Lower Intake** | **Moderate Intake** | **Higher Intake** |
| Model 3^c^ | Reference | 0.947 (0.895 – 1.002) | 1.015 (0.960 – 1.074) |

c; adjusted for the covariates in Model 2 and intakes of (a) processed meat, red meat, poultry, fish, dairy, refined grains, coffee, tea, soft drinks, and sugar and confectionary when plant-sourced nitrate or nitrite were the exposures of interest, and (b) vegetables, fruits, wholegrains, refined grains, coffee, tea, soft drinks, and sugar and confectionary when animal-sourced nitrate or nitrite were the exposures of interest., *; p < 0.05, **; p < 0.001.

**Table S16.** Fully adjusted cross-sectional analysis of the nitrate/nitrite intakes and Odds (95% CI) of periodontal disease in the UK Biobank study when including the Eatwell Guide score as a covariate (n=158778).

| Total Plant-Based Nitrate | | | |
| --- | --- | --- | --- |
|  | **Lower Intake** | **Moderate Intake** | **Higher Intake** |
| Model 3^c^ | Reference | 0.953 (0.922 – 0.986) * | 0.945 (0.913 – 0.979) * |
| Total Animal-Based Nitrate | | | |
|  | **Lower Intake** | **Moderate Intake** | **Higher Intake** |
| Model 3^c^ | Reference | 0.973 (0.942 – 1.005) | 1.009 (0.977 – 1.042) |
| Total Plant-Based Nitrite | | | |
|  | **Lower Intake** | **Moderate Intake** | **Higher Intake** |
| Model 3^c^ | Reference | 0.880 (0.854 – 0.906) ** | 0.860 (0.824 – 0.897) ** |
| Total Animal-Based Nitrite | | | |
| (mg/d) | **Lower Intake** | **Moderate Intake** | **Higher Intake** |
| Model 3^c^ | Reference | 0.986 (0.955 – 1.019) | 1.049 (1.016 – 1.084) * |

c; adjusted for the covariates in Model 2 and intakes of (a) processed meat, red meat, poultry, fish, dairy, refined grains, coffee, tea, soft drinks, and sugar and confectionary when plant-sourced nitrate or nitrite were the exposures of interest, and (b) vegetables, fruits, wholegrains, refined grains, coffee, tea, soft drinks, and sugar and confectionary when animal-sourced nitrate or nitrite were the exposures of interest., *; p < 0.05, **; p < 0.001.
